# Illinois State Medical Society

**Published:** 1857-05

**Authors:** 


*Illinois State Medical Society.*

H. NOBLE, M.D., McLean County, *President.*

C. N. ANDREWS, M.D., Rockford; F. R. PAYNE, M.D., Marshall; S. T. TROWBRIDGE, M.D., Decatur— *Committee of Practical Medicine.*

*Sir,*—The Committee of Practical Medicine of the Illinois State Medical Society are required by the Constitution of the Society to report annually "The more important improvements effected in this State in the management of individual diseases; and on the progress of epidemics, referring as occasion requires to Medical Topography and to the character of prevailing diseases in special localities." To enable the Committee to discharge this important duty, they respectfully ask your assistance in furnishing the information necessary for such a report. Answers to the following questions, or to any of them, forwarded to Rockford, directed to the Chairman of the Committee, by the 15th of May, will be thankfully received and properly credited.

For the purpose of continuing the very interesting and profitable discussion on the subject of Fever, which has taken place at the last two or three Annual Meetings of the Society, a few interrogatories relating specially to important points connected with the subject have been presented. And the Chairman of the Committee, having had occasion to note some observations, which, so far as they go, seem to show that children are not as liable to have Croup after having the Measles as before, and also that the membranous substance found lining the air passages in fatal cases of Croup, is not the result of any peculiar, pre-existing inflammation of the parts to which it is directly attached, but that it may be produced by severe and continued Dyspnœa from any cause situated within the Larynx, has also added a few interrogatories relating to these subjects:—

What has been the prevailing diseases in your neighborhood within the past year?

What was the season of their occurrence, and what the meteorological conditions at the time?

Was there anything peculiar in the character of those diseases?

What treatment appeared to be most successful?

Have any of the ordinary forms of Fever prevailed in your locality?

Do you give Quinine in the remitting or continued forms of Fever, either with the view of arresting their progress, or for the purpose of changing any usual or occasional conditions of the nervous or vascular system which occur in these forms of Fever? If so, please state your practice.

Have you observed any individual constitutional peculiarities, or any peculiar diathesis, in which Quinine cannot safely be resorted to as a febrifuge?

Have you observed that certain conditions of the brain in Fever contra-indicate the use of Quinine? If so, what are those conditions? by what symptoms do you recognize them? and what are the pernicious effects of Quinine when given when they are present?

Do you give mercury in Fever? If so, please state what indications you wish to answer in its use, and what symptoms or conditions indicate or contra-indicate its use.

Do you give Opium as a direct curative agent in Fevers? If so, what symptoms indicate its use? and by what rules are you governed in its administration?

Do you ever bleed in Fevers? If so, to answer what indication?

Do you ever attempt to arrest the progress of Fevers by the use of Emetics? If so, please state your method of using them, your precautions in their use, and what, in your opinion, is the *modus operandi* of their curative effect.

If you have any other methods of arresting the progress of Fever, not referred to above, please state them.

What vital organs have you observed to suffer most frequently in Fevers? and what has been the nature of these 'local complications,' and what their comparative effect upon the termination of the cases in which they have occurred?

Where, in your opinion, does 'death begin' in fatal cases of Fever? In other words, what vital functions usually first fail in Fevers, and what is the more usual immediate cause of death?

Did you ever know a case of Fever to terminate fatally in which there was not an evident destruction of some vital function, the result of local disease of some kind, which was the immediate cause of such fatal termination?

Have any children, in your knowledge, had well marked Croup, (as distinguished from Laryngitis, Edema of the Larynx, Spasmodic Croup, Ulceration of the Fauces and Larynx, with granular crusts or exudations, such as sometimes occurs in Scarlatina and Measles, producing Croupy symptoms,) who had previously had Measles, and if so, what proportion of cases proved fatal?

Have you any facts bearing upon the question that the membranous exudation or secretion seen in fatal cases of Croup, lining the air passages, is not the result of any primary inflammation of the Bronchial Membrane, but that it is produced after severe Dyspnœa—commences from some form of laryngial disease, and is the direct consequence of this Dyspnœa, and not the cause: the powerful but nearly ineffectual efforts at dilating the Chest producing sanguineous engorgement of, and fibrinous exudation from the Mucous Membrane, the same as if an exhausted Cupping Glass were applied to its surface?

Have you any practical suggestions to make to the Profession relating to the treatment of any disease? If so, please make them in detail. Please give, also, any Statistics you may have, relating to the subjects of the Committee's Report.

C. N. ANDREWS, M.D.,
ROCKFORD, WINNEBAGO COUNTY, ILLINOIS,
*Chairman of the Committee of Practical Medicine of the Illinois State Medical Society.*

---

*Illinois State Medical Society,*

Will meet in Chicago on the first Tuesday in June. Delegates and members, on arriving in the city, may find the place of meeting by calling on the Secretary, No. 53 Randolph St. Chicago.

